# Identification of hemagglutinin structural domain and polymorphisms which may modulate swine H1N1 interactions with human receptor

**DOI:** 10.1186/1472-6807-9-62

**Published:** 2009-09-28

**Authors:** Veljko Veljkovic, Henry L Niman, Sanja Glisic, Nevena Veljkovic, Vladimir Perovic, Claude P Muller

**Affiliations:** 1 Center for Multidisciplinary Research, Institute of Nuclear Sciences VINCA, P.O. Box 522, 11001 Belgrade, Serbia; 2 Recombinomics, Inc., 648 Field Club Road, Pittsburgh, PA 15238, USA; 3 Institute of Immunology Laboratoire National de Santé/CRP-Santé, Luxembourg, 20A rue Auguste Lumière, L-1950 Luxembourg, Grand-Duchy of Luxembourg

## Abstract

**Background:**

The novel A/H1N1 influenza virus, which recently emerged in North America is most closely related to North American H1N1/N2 swine viruses. Until the beginning of 2009, North American swine H1N1/N2 viruses have only sporadically infected humans as dead-end hosts. In 2009 the A/H1N1 virus acquired the capacity to spread efficiently by human to human transmission. The novel A/H1N1 influenza virus has struck thousands of people in more than 70 countries and killed more than 140, representing a public health emergency of international concern. Here we have studied properties of hemagglutinin of A/H1N1 which may modulate virus/receptor interaction.

**Results:**

Analyses by ISM bioinformatics platform of the HA1 protein of North American swine H1N1/N2 viruses and the new A/H1N1 showed that both groups of viruses differed in conserved characteristics that reflect a distinct propensity of these viruses to undergo a specific interaction with swine or human host proteins or receptors. Swine H1N1/N2 viruses that sporadically infected humans featured both the swine and the human interaction pattern. Substitutions F71S, T128S, E302K, M314L in HA1 of swine H1N1 viruses from North America are identified as critical for the human interaction pattern of A/H1N1 and residues D94, D196 and D274 are predicted to be "hot-spots" for polymorphisms which could increase infectivity of A/H1N1 virus. At least one of these residues has already emerged in the A/H1N1 isolates from Spain, Italy and USA. The domain 286-326 was identified to be involved in virus/receptor interaction.

**Conclusion:**

Our results (i) contribute to better understanding of the origin of the novel A/H1N1 influenza virus, (ii) provide a tool for monitoring its molecular evolution (iii) predicts hotspots associated with enhanced infectivity in humans and (iv) identify therapeutic and diagnostic targets for prevention and treatment of A/H1N1 infection.

## Background

Sporadic infections of humans by swine influenza viruses have been reported from the United States and worldwide, mostly from classical swine influenza [1-3]. During the late nineties multiple subtypes of triple reassortants influenza viruses with genes from avian, human and pig origin emerged and became predominant in North American swine [1,4]. Triple reassortant H1N1 and H1N2 subtypes occasionally infected humans but human to human transmission was rare and always very limited. However, disease severity and clinical out-come was always unpredictable [5-7]. In April 2009 a H1N1 triple reassortant swine influenza virus infected humans in North America [4] and continued to effectively transmit from human to humans (REF). The virus spread rapidly within Mexico and with some delay across the United States before spread to other continents. From 19 April to 1 August 2009, 60 655 specimens tested positive for influenza and were reported to Flu-Net by 73 countries, areas and territories [8]. As a result, WHO declared the virus a pandemic threat. While most fatal cases occurred in Mexico and the United States, the virus appeared to be less aggressive in Europe and Asia. The rapid spread of this swine influenza virus mainly among young healthy adults and outside of the classical influenza season added to the unpredictability of this virus. Thus the virus and its molecular evolution raise a number of questions that are of prime international public health concern.

Recently, we applied the Informational Spectrum Method (ISM) bioinformatics platform [9], for analysis of the structure and function of the HA subunit 1 (HA1) of H5N1 influenza viruses. Results of this analysis revealed that HA1 of H5N1 viruses encodes specific and highly conserved information which may determine the recognition and targeting of these highly pathogenic avian influenza (HPAI) viruses to their receptor [10]. We also showed that a subset of H5N1 in Egypt may be evolving toward an H1N1-like receptor usage, indicating more efficient human-to-human transmission [10]. This prediction is in accord with recently observed H5N1 subclinical cases in Egypt. This silent spread of H5N1 in human populations sets the stage for increased transmission efficiencies and represents another threat with pandemic potential.

Here we used the ISM platform to compare North American swine H1N1/N2 influenza viruses with the new pandemic A/H1N1 virus. Our results showed that both groups of viruses differed in conserved characteristics that reflect a distinct propensity of these viruses to undergo a specific interaction with swine or human host proteins or receptors. Swine H1N1/N2 viruses that sporadically infected humans featured both the swine and the human interaction pattern. Furthermore, we identified several amino acid positions that are predicted to be "hot-spots" for polymorphisms which could increase human infectivity of A/H1N1 virus. At least one of these residues has already emerged in the A/H1N1 isolates from Spain, Italy and USA.

## Methods

### Phylogenetic analysis

The tree was calculated with the Neighbour-Joining method (Kimura-2-parameter) using MEGA 4 software.

### Sequences

All HA1 sequences were retrieved from GenBank database. For the analysis of swine H1N1 and H1N2 influenza viruses found in North America between 1931 and 2008 all sequences were downloaded from GenBank:

A/Swine/Minnesota/55551/00 (H1N2) [AF455678]; A/Swine/Indiana/P12439/00 (H1N2) [AF455680]; A/Swine/Ohio/891/01(H1N2) [AF455675]; A/Swine/North Carolina/93523/01 (H1N2) [AF455677]; A/Swine/North Carolina/98225/01(H1N2) [AF455676]; A/Swine/Illinois/100084/01 (H1N2) [AF455682]; A/Swine/Illinois/100085A/01 (H1N2) [AF455681]; A/Swine/Iowa/930/01(H1N2) [AF455679]; A/SW/CO/17871/01(H1N2) [AY060046]; A/SW/MO/1877/01(H1N2) [AY060049]; A/SW/MN/23124-S/01(H1N2) [AY060048];/SW/MN/17138/01(H1N2) [AY060052]; A/Swine/Indiana/P12439/00 (H1N2) [AF455680]; A/Swine/Indiana/9K035/99 (H1N2) [AF250124]; A/swine/Minnesota/1192/2001(H1N2) [EU139828]; A/Swine/Ohio/891/01(H1N2) [AF455675]; A/swine/Guangxi/17/2005(H1N2) [EF556201]; A/SW/MN/23124-T/01(H1N2) [AY060047]; A/SW/MN/16419/01(H1N2) [AY060050]; A/SW/MN/23124-S/01(H1N2) [AY060048]; A/Swine/Illinois/100085A/01 (H1N2) [AF455681]; A/Swine/Illinois/100084/01 (H1N2) [F455682]; A/swine/Minnesota/00194/2003(H1N2) [EU139830]; A/swine/Kansas/00246/2004(H1N2) [EU139831]; A/swine/OH/511445/2007(H1N1) [EU604689]; A/Swine/North Carolina/93523/01 (H1N2) [AF455677]; A/swine/Memphis/1/1990(H1N1)) [CY035070]; A/swine/Maryland/23239/1991(H1N1)) [CY022477]; A/swine/California/T9001707/1991(H1N1)) [CY028780]; A/swine/Iowa/24297/1991(H1N1) [CY027155]; A/swine/Ohio/C62006/06(H1N1) [EU409960]; A/swine/Ohio/24366/07(H1N1) [EU409948]; A/swine/Iowa/17672/1988(H1N1) [CY022333]; A/swine/Wisconsin/1915/1988(H1N1) [CY022429]; A/swine/Iowa/31483/1988(H1N1) [CY022970]; A/Swine/Indiana/1726/1988(H1N1) [M81707]; A/swine/Kansas/3024/1987(H1N1) [CY025010]; A/swine/Kansas/3228/1987(H1N1) [CY022469]; A/swine/Iowa/2/1985(H1N1); [CY027507]; A/swine/Iowa/3/1985(H1N1) [CY022325]; Awine/Iowa/1/1985(H1N1) [CY022317]; A/swine/Iowa/1/1987(H1N1) [CY022962]; A/swine/Iowa/1/1986(H1N1) [CY028788]; A/swine/Tennessee/82/1977(H1N1) [CY022301]; A/swine/Minnesota/5892-7/1979(H1N1) [CY022365]; A/swine/Tennessee/4/1978(H1N1) [CY028427]; A/swine/Wisconsin/641/1980(H1N1) [CY022445]; A/swine/Wisconsin/629/1980(H1N1) [CY022994]; A/swine/Tennessee/87/1977(H1N1) [CY024970]; A/swine/Iowa/2/1987(H1N1) [CY028171]; A/swine/Wisconsin/663/1980(H1N1) [CY024994];A/swine/Wisconsin/661/1980(H1N1) [CY022453]; A/swine/Tennessee/84/1977(H1N1) [CY024954]; A/swine/Tennessee/88/1977(H1N1) [CY024978]; A/swine/Ontario/11112/04(H1N1) [DQ280250]; A/swine/Tennessee/5/1978(H1N1) [CY027515]; A/swine/Minnesota/37866/1999(H1N1) EU139827]; A/swine/Iowa/00239/2004(H1N1) [EU139832]; A/Swine/Wisconsin/457/98(H1N1) [AF222034]; A/Swine/Wisconsin/168/97(H1N1) [AF222031]; A/Swine/Wisconsin/163/97(H1N1) [AF222028]; A/Swine/Wisconsin/166/97(H1N1) [AF222030]; A/Swine/Wisconsin/458/98(H1N1) [AF222035]; A/Swine/Wisconsin/136/97(H1N1) [AF222027]; A/Swine/Wisconsin/164/97(H1N1) [AF222029]; A/Swine/Wisconsin/238/97(H1N1) [AF222033]; A/Swine/Wisconsin/235/97(H1N1) [AF222032]; A/Swine/Wisconsin/464/98(H1N1) [AF222036]; A/Swine/Wisconsin/125/97(H1N1) [AF222026]; A/swine/Iowa/1976/1931(H1N1)) [U11858]; A/swine/OH/511445/2007(H1N1) [EU604689].

### Informational spectrum method

It has been proposed that the number of valence electrons and the electron-ion interaction potential (EIIP) representing the main energy term of valence electrons are essential physical parameters determining of the long-range properties of biological molecules [11]. The EIIP can be determined for organic molecules by the following simple equation derived from the "general model pseudopotential" [12,13]:

(1)

where Z* is the average quasivalence number (AQVN) determined by

(2)

where Z_*i *_is the valence number of the *i*-th atomic component, *n*_*i *_is the number of atoms of the *i*-th component, *m *is the number of atomic components in the molecule, and Nis the total number of atoms. The EIIP values calculated according to equations (1) and (2) are in Rydbergs (Ry).

The EIIP parameter was used as a basis of the informational spectrum method (ISM) for structure/function analysis of proteins, analysis of protein - protein interaction and *de novo *design of biologically active peptides [for reviews see Ref. [9] and references therein]. Here we will only briefly present this bioinformatics method.

A sequence of N residues is represented as a linear array of N terms, with each term given a weight. The weight assigned to a residue is its EIIP value (given in Ry), determining electronic properties of amino acids, which are responsible for their intermolecular interactions (Table 1). In this way the amino acid sequence is transformed into a sequence of numbers. This numerical sequence, representing the primary structure of the protein, is then subjected to a discrete Fourier transformation, which is defined as follows:

**Table 1:** The electron-ion interaction potential (EIIP) of amino acids.

**Amino acid**	**EIIP [Ry]**
Leu	0.0000
Ile	0.0000
Asn	0.0036
Gly	0.0050
Glu	0.0057
Val	0.0058
Pro	0.0198
His	0.0242
Lys	0.0371
Ala	0.0373
Tyr	0.0516
Trp	0.0548
Gln	0.0761
Met	0.0823
Ser	0.0829
Cys	0.0829
Thr	0.0941
Phe	0.0946
Arg	0.0959
Asp	0.1263

(3)

where x(m) is the m-th member of a given numerical series, N is the total number of points in this series, and X(n) are discrete Fourier transformation coefficients. These coefficients describe the amplitude, phase and frequency of sinusoids, which comprised the original signal. The absolute value of a complex discrete Fourier transformation defines the amplitude spectrum and the phase spectrum. The complete information about the original sequence is contained in both spectral functions. However, in the case of protein analysis, relevant information is presented in an energy density spectrum, which is defined as follows:

(4)

In this way, sequences are analyzed as discrete signals. It is assumed that their points are equidistant with the distance d = 1. The maximal frequency in a spectrum defined as above is F = 1/2 d = 0.5. The frequency range is independent of the total number of points in the sequence. The total number of points in a sequence influences only the resolution of the spectrum. The resolution of the N-point sequence is 1/n. The n-th point in the spectral function corresponds to a frequency f(n) = nf = n/N. Thus, the initial information defined by the sequence of amino acids can now be presented in the form of the informational spectrum (IS), representing the series of frequencies and their amplitudes.

The IS frequencies correspond to the distribution of structural motifs with defined physicochemical properties determining a biological function of a protein. When comparing proteins, which share the same biological or biochemical function, the ISM technique allows detection of code/frequency pairs which are specific for their common biological properties, or which correlate with their specific interaction. These common informational characteristics of sequences are determined by cross-spectrum or consensus informational spectrum (CIS). A CIS of N spectra is obtained by the following equation:

(5)

where Π(i,j) is the j-th element of the i-th power spectrum and C(j) is the j-th element of CIS. Thus, CIS is the Fourier transform of the correlation function for the spectrum. Thus, any spectral component (frequency) not present in all compared informational spectra is eliminated. Peak frequencies in CIS are common frequency components for the analyzed sequences. A measure of similarity for each peak is a signal-to-noise ratio (S/N), which represents a ratio between signal intensity at one particular IS frequency and the main value of the whole spectrum. If one calculates a CIS for a group of proteins, which have different primary structures, and finds strictly defined peak frequencies, it means that the primary structures of the analyzed proteins encode the same information. It has been demonstrated that: 1) such a peak exists only for a group of proteins with the same biological function; 2) no significant peaks exist for biologically unrelated proteins; 3) peak frequencies are different for different biological functions. Furthermore, it was shown that the proteins and their targets (ligand/receptor, antibody/antigen, etc.) have the same characteristic frequency in common [9]. Thus, it can be postulated that IS frequencies not only characterize general function but also recognition and interaction between a particular protein and its target. Once the characteristic frequency for a particular protein function/interaction is identified, it is possible then to utilize the ISM approach to predict the amino acids in the sequence, which essentially contribute to this frequency and are likely to be crucial for the observed function [9]. The server for free on-line ISM analysis can be accessed at  and [14].

## Results and discussion

**Figure 1:**
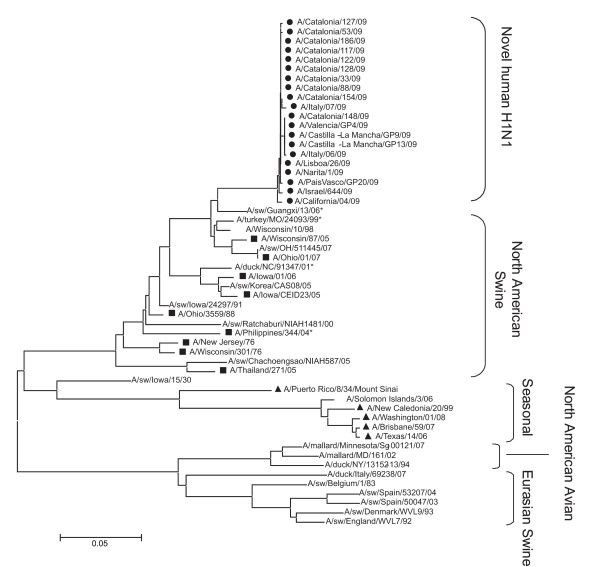
**Phylogeny of HA gene sequences of H1N1 and H1N2 viruses infecting humans**. Human cases of H1 swine that infected humans between 1976 and 2007 (black square); avian species (H1); seasonal human H1N1 influenza (black triangle), representative strains of Novel A/H1N1 influenza and H1N2 viruses (asterisk).

**Figure 2:**
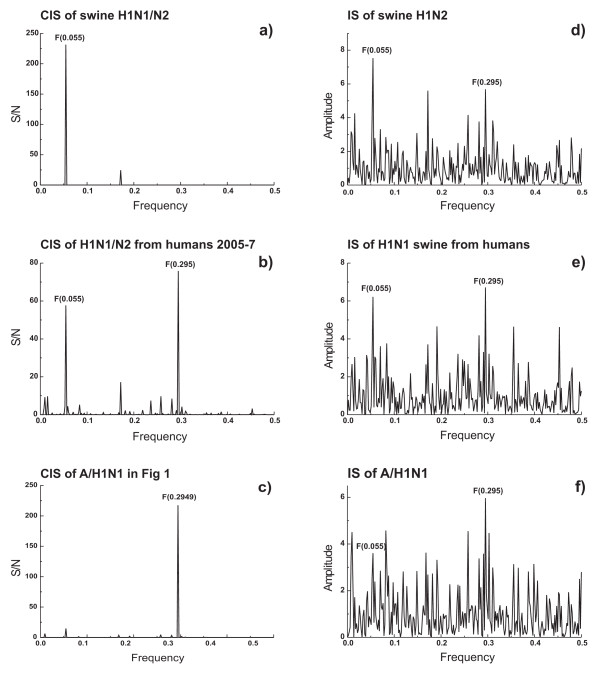
**The ISM analysis of HA1 proteins**. (a) CIS of swine H1N1 and H1N2 influenza viruses found in North America between 1931 and 2008, (b) CIS of swine H1N1 viruses infecting humans in US during 2005-2007, A/Iowa/01/2006; A/Wisconsin/87/2005; A/Ohio/01/2007; A/Ohio/02/2007, (c) CIS of A/H1N1 viruses presented in Figure 4. (d) IS of a representative swine H1N2 HA1 (A/swine/Minnesota/1192/2001), (e) IS of a representative swine H1N1 HA1 infecting human (A/Iowa/01/2006), and (f) IS of a representative A/H1N1 HA1 (A/Castilla-La Mancha/GP13/2009).

Phylogeny and principal component cluster analysis revealed that the H gene of A/H1N1 is most closely related to the triple reassortants swine influenza H1N1 and H1N2 found in North America since 2000 (Figure 1). These results, suggesting that A/H1N1 emerged from this cluster of viruses, are consistent with the recently reported findings of Trifonov and co-workers [15]. The H genes of swine H1N1 and H1N2 from North America were analyzed by the ISM and compared to A/H1N1 strains. Figure 2 shows the typical IS profile of a selected virus (Figure 2d, e, f) as well as the cumulated CIS (Figure 2a, b, c) of all viruses of each group.

The CIS of HA1 of swine H1N2/H1N1 and A/H1N1 strains have characteristic dominant peaks at the IS frequencies F(0.055) and F(0.295), respectively (Figure 2a and 2c). According to the ISM concept this reflects a differential interaction pattern of the HA1 of the two groups of viruses. The tropism of the viruses suggests that a high amplitude at F(0.055) corresponds to a higher propensity to interact with swine protein(s) while the high amplitude at F(0.295) may correspond to a preferred interaction with human protein(s). CIS of the HA1 gene of swine H1N1 viruses isolated from humans in US (before 2008) contains characteristic peaks at both frequencies F(0.055) and F(0.295) (Figure 2b). This suggests that these viruses, that sporadically infected humans, display both the distinct "swine" interaction pattern shared with the swine H1N1/N2 viruses and the characteristic "human" interaction pattern shared with A/H1N1 viruses. Thus, these three groups of viruses have a distinct propensity to interact with swine and human proteins which can be described by ISM analysis. These results also provide additional strong evidence that HA1 from swine viruses infecting humans in the US before 2008 were the likely precursors of A/H1N1.

Changes in H1N1/N2 viruses that lead to enhanced transmission in humans are of particular interest. The comparison of HA1 sequences of H1N1/N2 viruses which infected only swine with the early Mexican A/H1N1 strain A/Mexico/4115/2009 revealed 14 amino acid substitutions which are highly specific for A/H1N1 viruses (Table 2).

**Table 2:** Effect of HA1 polymorphisms on the amplitudes corresponding to IS frequencies F(0.055) and F(0.295).

**Mutation**	**ΔA [F(0.055)]** **(%)**	**ΔA [F(0.295)]** **(%)**	**Propensity for human interaction pattern**
R36K*	-7.5	+5.0	+
L61I	0	0	0
F71S	-1.2	+0.4	+
N97D	-6.6	-9.1	±
T128S	-1.4	+1.2	+
R130K	-5.1	-5.0	±
R146K	-7.5	-2.9	±
N168D*	+4.9	-4.8	-
T216I*	-5.7	+10.5	+
A224E	0	0	0
S271P*	-4.3	+2.7	+
V298I	0	0	0
E302K	-3.9	+1.7	+
M314L	-3.6	+6.5	+

Mutations present in swine H1N1/N2 strains which infect humans are marked with asterisk.

We further investigated which of these mutations or combinations thereof are most important for the switch between interaction patterns from swine H1N1/N2 to A/H1N1 strains. As shown above, the interaction between H1N1/N2 and swine protein(s), and the interaction between A/H1N1 and human protein(s) are characterized by the frequencies F(0.055) and F(0.295), respectively. According to the ISM concept [9,16] mutations in HA1 which increase the amplitude at F(0.295) and decrease amplitude at F(0.055) would potentially contribute to the switch of the viral host tropism from swine to human. Seven of the 14 mutations presented in Table 2 (R36K, F71S, T128S, T216I, S271P, E302K, M314L) increase amplitude on the F(0.295) and decrease amplitude on F(0.055), suggesting that these mutations may be critical for the switch of H1N1/N2 from a swine to a human tropism. It is of note that three of these mutations (R36K, T216I, S271P) also are present in swine H1N1 viruses that infected humans in the US between 2005 and 2007 (Figure 2b). ISM analysis also showed that any of the combinations of the mutations F71S, T128S, E302K and M314L, that are only present in A/H1N1, decrease the amplitude in F(0.055) and increase the amplitude in F(0.295). This suggests that these four mutations may play an important role in the efficient infection of humans by A/H1N1, and perhaps the effective human to human transmission. It is of interest to note that 7 of 14 mutations presented in Table 2 decrease amplitude on F(0.055) and increase amplitude on F(0.295), 6 mutations decrease amplitudes on both frequencies or have no effect, and only one (N168D) increases the amplitude on F(0.055) and decreases amplitude on F(0.295). This suggests that the mutations in A/H1N1 that predispose for the human interaction pattern are remarkable of more prevalent than mutations that predispose for the swine interaction pattern.

**Figure 3:**
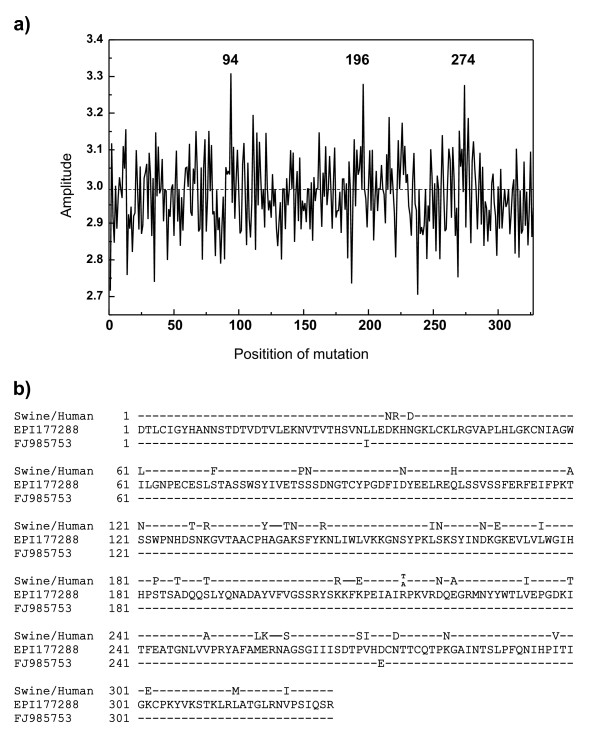
**Identification of hot-spots for mutations that may increase A/H1N1/-receptor interaction**. (a) Effect of alanine substitution on the amplitude at frequency F(0.295). "In silico" alanine scan of the complete HA1 sequence of the early A/H1N1 isolate A/Mexico/4115/2009 (EPI177288). (b) Homology between HA1 of A/Castilla-La Mancha/GP13/2009 (FJ985753) (identical to US isolate A/South Carolina/09/2009), swine H1N1 viruses infecting human 2005-2007 and A/Mexico/4115/2009 (EPI177288).

**Figure 4:**
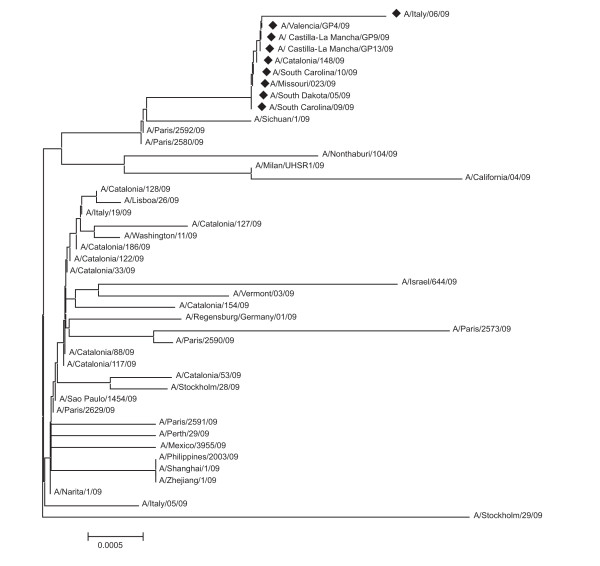
**Phylogeny of HA gene sequences of representative strains of Novel H1N1 influenza**. Strains with specific D274E mutations (black diamond).

It can be expected that A/H1N1 strains will accumulate additional polymorphisms in their HA1 genes which further favor the "human" interaction pattern and according to the ISM concept would be associated with an increase of the amplitude at frequency F(0.295) and decrease the amplitude at F(0.055). To identify candidate residues ("hot-spots") for such polymorphisms, we performed an *in silico *alanine scan of the complete HA1. This analysis revealed that mutations of residues 94D, 196D and 274D would increase the amplitude at the critical frequency F(0.295) (Figure 3a). Since Asp has the highest EIIP value (Table 1) substitutions in any of the above positions will increase the amplitude at frequency F(0.295). Interestingly, Asp (single letter code D) in positions 94, 196 and 247 is highly conserved in all North American swine H1N1/N2 strains and in all A/H1N1 HA1 genes. The only notable exceptions are four A/H1N1 isolates from Spain (A/Castilla-La Mancha/GP13/2009, A/Castilla-La Mancha/GP9/2009, A/Valencia/GP4/2009, A/Catalonia/P148/2009), two isolates from Italy (A/Italy/06/2009) and four isolates from US (A/South Carolina/09/2009; A/South Dakota/05/2009; A/South Carolina/10/2009; A/Missouri/023/2009) that have a D274E mutation (Figure 4). This mutation significantly increases the amplitude on the frequency F(0.295) and probably enhances the "human" interaction pattern. The same result was obtained for scan with any other amino acid with exception of Asp (results not shown). As can be seen in Figure 3b, A/Castilla-La Mancha/GP13/2009 (FJ985753) which is identical to A/South Carolina/09/2009 (GQ221794) differ from the early Mexican A/H1N1 isolates A/Mexico/4115/2009 (EPI177288) only in positions I32L and E257D. The amino acids L and I have the same EIIP value (see Table 1) and the L>I substitution does not affect the informational spectrum. In contrast, the EIIP values of amino acids D and E are significantly different (see Table 1) and the D>E mutation increases the amplitude at frequency F(0.295) by 15%, with little effect on structural properties of the protein since both amino acids are negatively charged. The stable mutation D274E found in US, Spain and Italy may correspond to further adaptation of A/H1N1 to humans. Interestingly, Spain was also one of the first (European) countries with indigenous chains of transmission, at a time when in other countries the virus was mostly found in imported cases.

**Figure 5:**
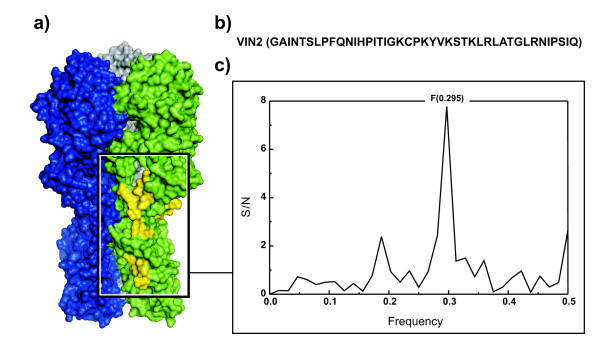
**A/H1N1 HA trimer and details of the VIN2 region**. The informational spectrum and position of the VIN2 domain (yellow) in the 3D structure of A/H1N1 isolate A/California/04/2009.

The computer scanning survey of the HA1 amino acid sequence of A/H1N1 strains showed that the main contribution to the information represented by the frequency F(0.295) comes from a domain located in the C-terminus of the protein which encompasses residues 286 - 326 (denoted VIN2) of the mature protein. Figure 5. shows IS and position of the VIN2 domain in the 3D structure of A/H1N1 isolate A/California/04/2009. It is of note that VIN2 is conserved in all A/H1N1 and that two of the four polymorphisms (E302K and M314L) which are identified as critical for human infection are located within this domain. The significance of these polymorphisms are strengthened by the fact that domain 286 - 326 also is highly conserved in HA1 of swine viruses (only 30 of 500 swine H1N1 HA1 presented in UniProt database have mutations in this domain).

The relative position of the receptor binding domain and the receptor targeting domain (VIN2) in 3D structure of A/H1N1 HA1 is similar to the position of these two domains in seasonal flu H1N1 viruses but different than in H1N1 1918 viruses [10]. This suggests that efficacy of interaction between A/H1N1 and its receptor is similar to seasonal flu H1N1 viruses but less efficient than in 1918 viruses.

## Conclusion

Analyses by the ISM bioinformatics platform of the HA1 protein of North American swine H1N1 and H1N2 viruses and the new A/H1N1 that emerged recently in Mexico and the USA showed that both groups of viruses differed in characteristic parameters that reflect a distinct propensity of these viruses to undergo a specific interaction with swine or human host proteins or receptors. Using the same approach, amino acid substitutions F71S, T128S, E302K, M314L in the A/H1N1 HA1 essential for the human interaction pattern of these viruses were identified and residues 94D, 196D and 274D of A/H1N1 HA1 were predicted as "hot-spots" for mutations that may significantly increase the propensity of this virus to interact preferentially with human host proteins. At least one of these mutations (D274E) was already found in the A/H1N1 isolates from Spain, Italy and US, suggesting the virus further adapts to the human host. In addition, it has been suggested that the highly conserved domain 286 - 326 of HA1 plays an important role in A/H1N1-receptor interaction and represents a candidate target for diagnostics, vaccines and therapies.

## Authors' contributions

VV conceived of the study, participated in its design and coordination and preparation of the manuscript, HLN carried out the molecular genetic study and drafted the manuscript, SG carried out mutation and structure/function analysis of viral proteins, NV carried out the ISM analysis of viral sequences. VP developed the ISM software for bioinformatics analysis of viral proteins and CPM performed phylogenetic and 3D structural analysis of viral proteins and participated in preparation of the manuscript. All authors read and approved the final manuscript.
